# Excessive early-life dietary exposure: a potential source of elevated brain iron and a risk factor for Parkinson’s disease

**DOI:** 10.1038/s41531-016-0004-y

**Published:** 2017-01-05

**Authors:** Dominic J Hare, Bárbara Rita Cardoso, Erika P Raven, Kay L Double, David I Finkelstein, Ewa A Szymlek-Gay, Beverley-Ann Biggs

**Affiliations:** 1 Department of Medicine (Royal Melbourne Hospital) at the Doherty Institute, The University of Melbourne, Parkville, Melbourne, VIC Australia; 2 The Florey Institute of Neuroscience and Mental Health, The University of Melbourne, Parkville, Melbourne, VIC Australia; 3 Elemental Bio-imaging Facility, University of Technology Sydney, Broadway, NSW Australia; 4 Department of Pharmaceutical Sciences, Department of Food and Experimental Nutrition, University of São Paulo, São Paulo, Brazil; 5 Center for Functional and Molecular Imaging, Georgetown University Medical Centre, Washington DC, USA; 6 Advanced Magnetic Resonance Imaging Section, Laboratory of Functional and Molecular Imaging, National Institute of Neurological Disorders and Stroke, National Institutes of Health, Bethesda, MD USA; 7 Sydney Medical School, University of Sydney, Darlington, NSW Australia; 8 Brain and Mind Centre, University of Sydney, Camperdown, NSW Australia; 9 Institute for Physical Activity and Nutrition, School of Exercise and Nutrition Sciences, Deakin University, Geelong, VIC Australia; 10 Victorian Infectious Diseases Service, Royal Melbourne Hospital, Parkville, Melbourne, VIC Australia

## Abstract

Iron accumulates gradually in the ageing brain. In Parkinson’s disease, iron deposition within the substantia nigra is further increased, contributing to a heightened pro-oxidant environment in dopaminergic neurons. We hypothesise that individuals in high-income countries, where cereals and infant formulae have historically been fortified with iron, experience increased early-life iron exposure that predisposes them to age-related iron accumulation in the brain. Combined with genetic factors that limit iron regulatory capacity and/or dopamine metabolism, this may increase the risk of Parkinson’s diseases. We propose to (a) validate a retrospective biomarker of iron exposure in children; (b) translate this biomarker to adults; (c) integrate it with in vivo brain iron in Parkinson’s disease; and (d) longitudinally examine the relationships between early-life iron exposure and metabolism, brain iron deposition and Parkinson’s disease risk. This approach will provide empirical evidence to support therapeutically addressing brain iron deposition in Parkinson’s diseases and produce a potential biomarker of Parkinson’s disease risk in preclinical individuals.

## Introduction

In their most recent guidelines for iron supplementation of infants and children, the World Health Organisation (WHO) states that a research priority should be the collection of ‘additional data on the safety of iron supplementation [including] effects in non-an[a]emic or non-iron-deficient children*’*.^1^ We acknowledge that iron fortification and supplementation programs have been a widely successful practice for reducing the incidence of iron deficiency anaemia (IDA), though we wish to highlight the importance of determining the optimal level of iron exposure to ensure IDA is avoided while also limiting potential negative health outcomes later in life. Ten-year follow-up studies of infants exposed to ‘off-the-shelf’ formula products with high (12.7 mg/L) iron content have suggested possible negative long-term developmental outcomes, justifying the need for studies of excessive early-life exposure in iron-replete populations with respect to adverse neurological effects. In this Perspective, we discuss the potential impact of long-standing iron fortification policies during periods of early-life brain development (which we consider as 0–24 months) on age-related iron accumulation and Parkinson’s disease (PD) risk.

### Dopamine and iron dyshomeostasis in Parkinson’s disease

Abnormally elevated iron within the midbrain beyond that of normal ageing^2^ is a pathological feature of PD.^3^ We have suggested that neurodegeneration observed in PD is initiated by aberrant reactions between redox-active iron and dopamine due to defective mechanisms regulating both chemicals. Cellular and animal studies have shown that prior neuronal iron accumulation (likely supplemented by glia and a neuronal labile iron pool that increases with age)^4^ overwhelms iron storage mechanisms, releasing it into the cytoplasm, where it interacts freely with dopamine to form toxic metabolites,^5^ and that iron levels within the vulnerable substantia nigra pars compacta region are elevated in the human PD brain.^6^

Overexpression of the key parkinsonian protein α-synuclein promotes neuronal iron accumulation,^7^ while iron itself can promote aggregation of mutated forms of this protein.^8^ Wild-type mice, aged normally after elevated iron exposure from 10 to 14 days (equivalent to our proposed critical window in humans of 6–24 months postpartum), that had elevated exposure comparable to iron-fortified infant formula had elevated nigral iron,^9^ increased oxidative stress markers and dopaminergic-specific neuron loss when assessed at 8 months of age, and a parkinsonian phenotype apparent from 5 months.^10^ The damage elicited by iron supplementation could be, in part, rescued using the low-affinity iron chelator clioquinol. However, when iron loading was combined with overexpression of the A53T mutated form of human α-synuclein associated with producing permeable dopamine-containing vesicles, the phenotype could not be recovered.^10^

Dyshomeostasis of iron and dopamine in PD is potentially hazardous. Numerous genetic mutations impair the function of α-synuclein, which is involved in vesicular packaging of dopamine.^11^ Permeabilised vesicles can leak dopamine into the cytosol, creating a neurotoxic iron-dopamine redox couple arising from concurrent genetic mishandling of normal iron storage and regulation.^12–14^ The reaction between dopamine and iron produces several highly neurotoxic metabolites of the catecholamine, which can eventually overwhelm endogenous antioxidant mechanisms that protect neurons against elevated oxidative load. Early-life exposure to high levels of iron may predispose the brain to increased deposition with age,^15^ which further stresses impaired genetic regulation of this essential metal. Polymorphisms have been identified in the transferrin gene,^13,14^ as well as increased expression of Ndfip1, a protein regulating the iron import protein divalent metal transporter-1, in the human substantia nigra.^16^ Parkinson-specific iron and dopamine metabolism deficits suggest that postnatal iron overload is unlikely to solely cause disease. Rather, a multi-hit hypothesis involving neuronal iron accumulation and defective genetic regulation of iron and dopamine metabolism may be more appropriate, the effects of which are exacerbated by excessive iron intake during critical windows of iron-dependent neurodevelopment. Additionally, multiple non-genetic risk factors, including coffee, alcohol and tobacco consumption, dietary habits, inflammatory disorders and occupational exposure to heavy metals (such as manganese) and pesticides, may have a compounding effect.^17^ For instance, iron and paraquat (as well as rotenone and the parkinsonian mimetic MPTP)^18^ exhibit a synergistic effect promoting neurotoxicity in sporadic PD.^19^ As with all epidemiological studies, particularly those investigating late-age-onset diseases like PD, discerning the direct effects of a single factor like early-life iron overload is extremely difficult.

### Parkinson’s mortality and historical iron fortification policies in high-income countries

We hypothesise that individuals exposed to high dietary iron during critical periods of neural development are at risk of excessive iron accumulation in the brain. For example, in high-income countries where cereals and infant formulae have historically been fortified with iron, infants may experience increased early-life iron exposure that predisposes them to age-related iron accumulation in susceptible neurons in the midbrain, which rapidly amasses iron during early life.^2^ Combined with genetic factors that limit iron-regulatory capacity and/or dopamine metabolism, this may increase the risk of PD.^20^

Given the association between brain iron and PD, we sought epidemiological evidence that high-income countries that were early adopters of food fortification and iron supplementation programs for infants may now be experiencing increasing rates of PD. Public health policies regarding dietary iron supplementation can be traced back nearly a century. The US began fortifying flour with iron in the 1930s to reduce IDA in the general population. This approach was reinforced by an American Medical Association policy in 1936^21^ that was adopted by most high-income countries during World War 2 and beyond, including countries receiving humanitarian aid from the US.^22^ Other than the US (wheat, maize and rice) and UK (wheat), where fortification remains mandatory, most other high-income countries either continue (e.g. Australia) or recently abandoned a voluntary fortification program (e.g. Sweden).^23^

Formula fortification to address IDA in infants was first endorsed by the American Academy of Paediatrics in 1969 and enacted into law in the Infant Formula Act (1980), though iron-enriched formulae were available from 1959.^24^ Current AAP recommendations suggest fortified formula with 12 mg/L of iron, in addition to complementary foods (including those manufactured from enriched grains and foods containing haem iron) from 6 months of age;^25^ most high-income countries follow this guideline to some degree. The European Society for Paediatric Gastroenterology, Hepatology, and Nutrition considers fortification of formula fed to infants from 6 months safe and appropriate; however, a specific level has not been set due to a cited lack of evidence for optimal iron levels to prevent IDA, and typical concentrations are 4–8 mg/L.^26^ When taken together, formula and iron-enriched cereal products account for a substantial portion of an infant’s dietary iron intake; in Australia this constitutes 71.7 % of the total iron intake for 9-month-old infants^27^ and 87.8 % for 6–12-month-old infants in the US.^28^

**Fig. 1:**
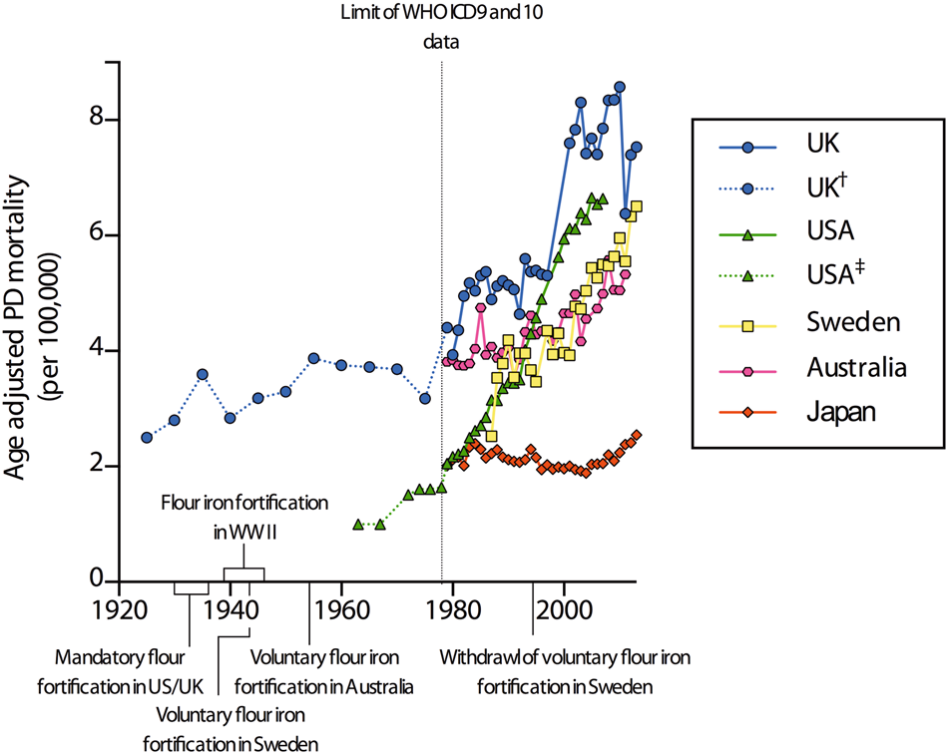
Age-adjusted mortality rates for PD in males and females from selected high-income countries. All data were obtained from the WHO mortality database (http://www.who.int/healthinfo/mortality_data/en/) and were age-adjusted to the year 2000 population for each country. Increasing PD mortality is most marked in the UK and USA approximately 50–65 years following initiation of mandatory iron fortification of grains and milled flour (1980–1995). Japan, with no policy on iron fortification, shows relatively stable mortality rates. PD as the cause of death was defined according to the ICD10 code G20 or ICD9 code 332 (both PD). Figures from the UK for 1984–1992 were corrected for dual listing of PD as a chronic condition and a cause of death reported by the Office of Population Censuses and Surveys to the WHO, as suggested by Clarke.^29^ Although WHO mortality data extend only to 1979, Clarke showed that age-adjusted PD mortality in the UK was relatively static in the decades preceding 1980 (†; dashed *blue line*).^29^ Pre-1979 mortality data for the US (‡) was obtained from Hinz *et al*.^30^ and Lilienfield *et al*.^31^ Note that these figures are estimates only and are not age-adjusted to the population at 2000. Mortality was used in place of prevalence or incidence rates to remove confounding effects of improving diagnosis or increasing disease duration though medical intervention, such as the introduction of L-DOPA treatment in the early 1980s

Age-adjusted mortality rates for PD (according to ICD9 and 10) in selected high-income countries show an increase from 50 years (the age from which PD incidence increases)^17^ post introduction of fortification policies (Fig. 1). This is highest in the US and UK, where fortification of grains remains mandatory. Intrapolation of data from alternative sources^29–31^ of PD mortality in the US and UK shows a comparatively static rate of mortality following the introduction of food fortification policies. Interestingly, Japan, which has no current or previous policy of mandatory nor voluntary iron fortification of grains, rice or infant formulae,^32^ is the primary exception as PD-associated mortality remains relatively low and static from 1979 to 2013. Genetic diversity with regard to known mutations impacting iron metabolism in PD may compound this feature. Interestingly, neither the PLA2G6 variant to *PARK14* ^33^ nor the ATP13A2 variant to *PARK9* ^34^ that have known association with iron metabolism and PD have been identified in large Japanese cohorts. A curiosity is observed from the limited PD WHO data for Norway, where PD mortality is relatively high (7.434 ± 0.810 per 100,000; 1996–2013) although iron fortification of common food products has been outlawed since the release of the first ‘White Paper’ on nutrition in 1976.^35^ However, infants are encouraged to be fed iron-fortified porridge since 1963,^36^ though a significant ‘lag time’ to demonstrate an effect on PD mortality has not yet elapsed. It is unclear if widespread fortified foods were available before 1976, which may contribute to high mortality rates. Norway does have disproportionately high rates of hereditary haemochromatosis^37^ that can result in basal ganglia iron deposition. There is conflicting evidence as to whether the C282Y, S65C and H63D mutations to the *HFE* gene are associated with PD;^38^ most recent statistically powered studies have suggested they are not.^39,40^

The increase in PD mortality we observed in the US is supported by recent data reported by Savica *et al.*,^41^ who showed an increase in PD prevalence occurring between 1976 and 2005 in a US-based cohort of 906 patients, particularly in males. The authors postulated a possible association between increased prevalence with declining smoking behaviour during this period, though acknowledged lifestyle and environmental factors may have contributed to this trend. It should be noted that sex-based differences in PD prevalence and mortality are controversial;^17^ it has been suggested that oestrogens increase striatal dopamine levels.^42^ While this likely prolongs disease duration in females, it is independent of potential increased risk from high iron exposure during early life.

The report by Savica *et al.*, combined with our assessment of PD mortality in high-income countries, supports the hypothesis that early-life iron exposure may increase PD risk in susceptible individuals. However, testing this hypothesis is a difficult task. Establishing a birth cohort to link early-life iron intake to age-related neurodegeneration in PD is impractical as it would not provide answers for 60–70 years. As prevalence of clinical PD dramatically increases above 65 years of age and is predicted to grow across all age groups in the future,^17^ the broader effects of the uptake of longstanding early-life iron fortification programs we observed from the late 1970s onwards may not be fully felt until 2030.

### Testing the link between early-life iron exposure and PD risk

**Fig. 2:**
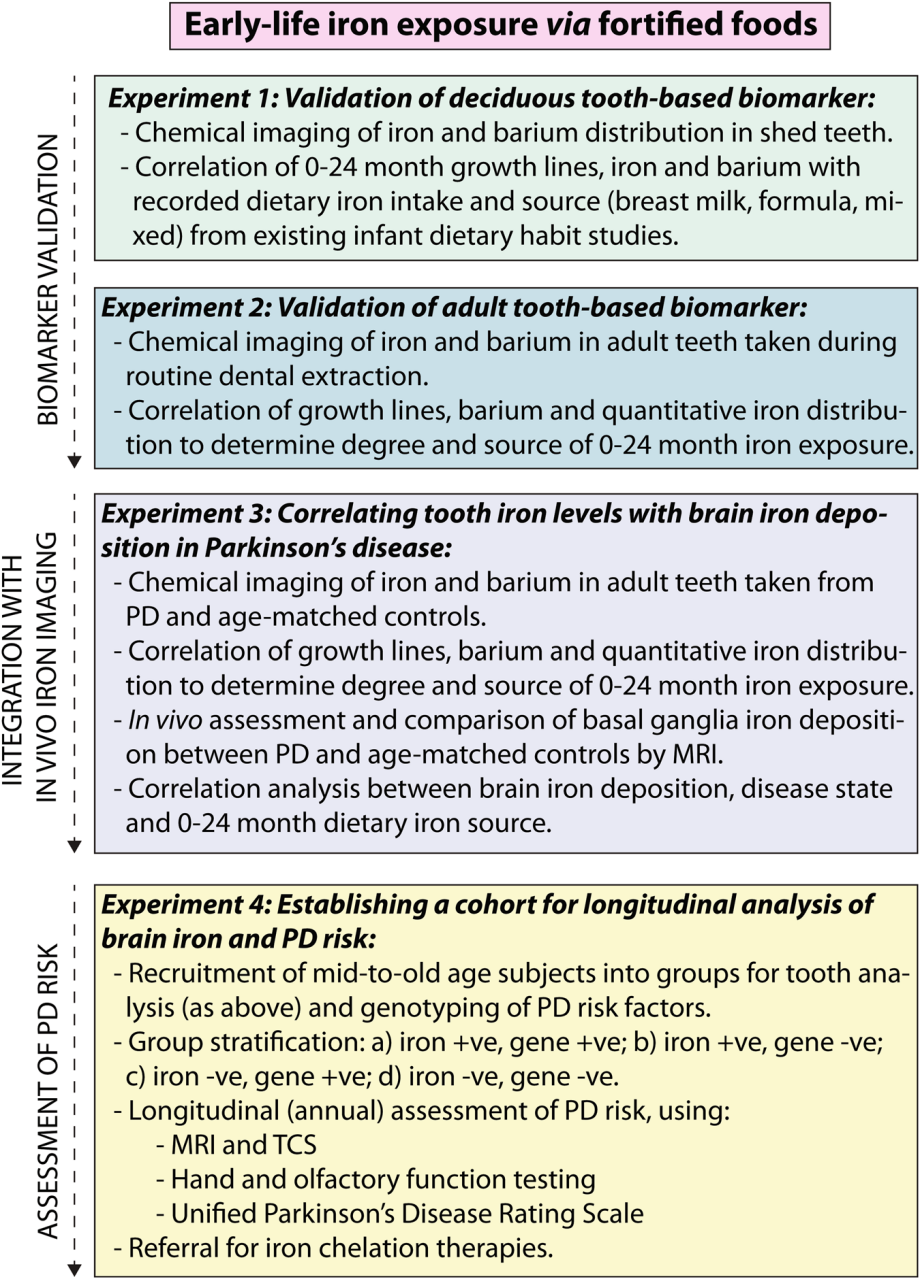
Flowchart for testing the early-life iron exposure hypothesis of PD risk. Biomarker validation*:* Two preclinical studies will be used to validate and apply a tooth-based biomarker of elevated dietary iron exposure during the 0–24-month period, using children’s deciduous teeth and adult teeth collected after routine dental extraction. Integration with in vivo brain iron imaging*:* Using the validated tooth biomarker, teeth collected from PD patients and age-matched controls will be analysed for temporal iron levels and source (via temporal analysis of barium levels)^43^ during the 0–24-month proposed critical window and assessed against iron deposition levels within the basal ganglia, measured by iron-specific MRI methods. Assessment of PD risk*:* A cohort of mid-to-old age subjects would be recruited to longitudinally assess for PD risk, based on stratification of (a) high dietary iron and presence of selected genetic risk factors (e.g. iron/dopamine metabolic regulators; the established PD genetic risk score); (b) high dietary iron exposure and no identified genetic risk factors; (c) low or normal early-life iron exposure with presence of known genetic risk factors; and (d) neither high iron nor identified genetic risk. Correlation analysis between each group with brain iron deposition (measured by MRI and TCS) and other preclinical measures of PD risk (e.g. hand and olfactory function and longitudinal multifactorial rating scales) will be performed. Followed longitudinally, evidence of increased PD risk in line with the preclinical diagnostic criteria set can be used to consider candidates for treatment with iron-chelating agents such as deferiprone^56^ to reduce brain iron burden and limit potential oxidative reactions in dopaminergic neurons mediated by iron prior to, or immediately following, onset of clinical symptoms

Blood iron levels provide only a relatively short window of exposure, and thus a retrospective biomarker of iron intake is needed. Humans retain a lifelong record of early-life iron exposure in teeth, which we propose as one of four consecutive experiments to test our hypothesis and translate our findings into a biomarker of PD risk (Fig. 2). Firstly, deciduous teeth must be established as a validated biomarker of early-life iron exposure. During odontogenesis, metals are retained in hydroxyapatite deposited in enamel and dentine. Using chemical microimaging, alignment of spatial metal distribution with developmental growth lines, akin to rings in a tree trunk, can be used to quantify temporal iron exposure at time scales equivalent to 1 week.^15^ Detailed dietary data, including iron intake, for accessible cohorts of infants can be repurposed to measure postnatal iron intake as the children reach the ages of 5–8 years when teeth are shed. Temporal growth lines and tooth iron concentrations can be correlated with recorded dietary iron intakes during the 6–24-month period. Further, spatial barium levels have been validated as a biomarker of dietary sources in early life, and correlation with iron distribution can differentiate between intake sources (i.e. exclusive breastfeeding vs. fortified formula and iron-enriched complementary feeding).^43^

This biomarker could then be applied to adult teeth to investigate associations between early-life iron intake and iron deposition in the ageing brain. Adult maxillary and mandibular first molars commence calcification at birth,^44^ and are therefore a record of iron exposure during the proposed critical window in early childhood. We suggest that adult teeth can be used to assess early-life iron intake and dietary source, and quantified using chemical microimaging without the need for deciduous teeth or patient recall. In this phase of testing our hypothesis, donated permanent teeth from healthy adults, individuals with clinically diagnosed PD and age-matched controls would be used to quantify early-life iron exposure, potential dietary sources, and to examine relationships with brain iron levels. Teeth are often extracted during routine dental procedures in older individuals; PD patients lose an average of 18 adult teeth, with age-matched controls losing 14, likely due to poor oral hygiene.^45^ Even in middle age, in the US the mean number of teeth lost or extracted during dentistry is approximately seven;^46^ tooth fragments obtained from dental crown implantation or partial extractions can also be used.

In vivo imaging assesses the degree of midbrain iron deposition and could be combined with a tooth biomarker of iron intake to investigate the aetiology of PD. Magnetic resonance imaging (MRI) techniques, including transverse relaxation rate (R_2_ or R_2_*) and quantitative susceptibility mapping, are highly sensitive to the paramagnetic effects of iron on T_2_* relaxation, an effect amplified at high magnetic field strengths. Several MRI studies have observed elevated iron in the PD substantia nigra;^47–49^ however, continued iron accumulation (which itself may be a useful tool for measuring PD progression) occurs over the disease duration.^50, 51^ Transcranial ultrasonography (TCS) also visualises increased iron-associated echogenicity within the substantia nigra in PD,^52^ is stable throughout disease duration^53^ and is of particular importance in detecting early biochemical and motor function changes.^54^

Finally, to test our proposed link between iron exposure during infancy and preclinical PD risk, we would establish an ‘at risk’ study cohort of mid to old-aged individuals. Combining in vivo imaging and genotyping of PD risk factors with our proposed tooth biomarker would provide an unbiased historical record of iron intake during the critical developmental window that imaging alone could not achieve. Here, we would perform chemical assessments of donated teeth and in vivo brain iron imaging, in addition to detecting mutations to iron and dopamine regulatory genes known to be associated with PD pathology.^12^ It is also important to consider possible confounding influences on circulating iron levels during early life, such as hereditary haemochromatosis. These groups could then be stratified by early-life iron exposure (i.e. ‘low’ and ‘high’ risk; measured via tooth analysis), genetic markers and other prodromal features, such as constipation, anxiety, depression and anosmia. Subjects could then be recruited for longitudinal population-based modelling of early PD detection,^55^ including testing of hand and olfactory function and longitudinal PD rating scales. Periodic in vivo brain iron assessment would project the trajectory of age-related iron deposition and correlate this with established preclinical features of PD (Fig. 2).

## Conclusions

A link between early-life dietary iron and PD would have major implications for public health policy, potentially prompting a review of iron fortification policies in countries in which IDA is no longer a public health threat. The preclinical period of PD is 8–17 years. PD patients have already experienced significant neurodegeneration at the time of diagnosis; therefore, identifying at-risk individuals or those with early-stage disease will increase opportunities to directly target the molecular basis of PD, mitigating or even preventing dopamine denervation prior to the onset of debilitating clinical symptoms. Empirical support for the use of the high-affinity iron chelator deferiprone in the early stages of clinical PD has been reported by Devos *et al.*,^56^ who used R_2_* MRI to confirm a reduction in brain iron and improvements in the Unified PD Rating Scale. This is now in phase II trials (ClinicalTrials.gov Identifier: NCT02728843).
